# Usefulness of Diastolic Function Score as a Predictor of Long-Term Prognosis in Patients With Acute Myocardial Infarction

**DOI:** 10.3389/fcvm.2021.730872

**Published:** 2021-09-10

**Authors:** SungA Bae, Hyun Ju Yoon, Kye Hun Kim, Hyung Yoon Kim, Hyukjin Park, Jae Yeong Cho, Min Chul Kim, Yongcheol Kim, Youngkeun Ahn, Jeong Gwan Cho, Myung Ho Jeong

**Affiliations:** ^1^Division of Cardiology, Department of Internal Medicine, Yonsei University College of Medicine and Cardiovascular Center, Yongin Severance Hospital, Yongin, South Korea; ^2^Division of Cardiology, Chonnam National University Hospital, Chonnam National University Medical School, Gwangju, South Korea

**Keywords:** diastolic function, left ventricular ejection fraction, myocardial infarction, mortality, heart failure

## Abstract

**Background:** Left ventricular diastolic function (LVDF) evaluation using a combination of several echocardiographic parameters is an important predictor of adverse events in patients with acute myocardial infarction (AMI). To date, the clinical impact of each individual LVDF marker is well-known, but the clinical significance of the sum of the abnormal diastolic function markers and the long-term clinical outcome are not well-known. This study aimed to investigate the usefulness of LVDF score in predicting clinical outcomes of patients with AMI.

**Methods:** LVDF scores were measured in a 2,030 patients with AMI who underwent successful percutaneous coronary intervention from 2012 to 2015. Four LVDF parameters (septal e′ ≥ 7 cm/s, septal E/e′ ≤ 15, TR velocity ≤ 2.8 m/s, and LAVI ≤ 34 ml/m^2^) were used for LVDF scoring. The presence of each abnormal LVDF parameter was scored as 1, and the total LVDF score ranged from 0 to 4. Mortality and hospitalization due to heart failure (HHF) in relation to LVDF score were evaluated. To compare the predictive ability of LVDF scores and left ventricular ejection fraction (LVEF) for mortality and HHF, receiver operating characteristic (ROC) curve and landmark analyses were performed.

**Results:** Over the 3-year clinical follow-up, all-cause mortality occurred in 278 patients (13.7%), while 91 patients (4.5%) developed HHF. All-cause mortality and HHF significantly increased as LVDF scores increased (all-cause mortality–LVDF score 0: 2.3%, score 1: 8.8%, score 2: 16.7%, score 3: 31.8%, and score 4: 44.5%, *p* < 0.001; HHF–LVDF score 0: 0.6%, score 1: 1.8%, score 2: 6.3%, score 3: 10.3%, and score 4: 18.2%, *p* < 0.001). In multivariate analysis, a higher LVDF score was associated with significantly higher adjusted hazard ratios for all-cause mortality and HHF. In landmark analysis, LVDF score was a better predictor of long-term mortality than LVEF (area under the ROC curve: 0.739 vs. 0.640, *p* < 0.001).

**Conclusion:** The present study demonstrated that LVDF score was a significant predictor of mortality and HHF in patients with AMI. LVDF scores are useful for risk stratification of patients with AMI; therefore, careful monitoring and management should be performed for patients with AMI with higher LVDF scores.

## Introduction

Acute myocardial infarction (AMI) is characterized by regional myocardial injury that may lead to systolic and diastolic dysfunction due to left ventricular (LV) remodeling and dysfunction. Left ventricular diastolic function (LVDF), an aftermath of AMI, is an important predictor for major adverse events (1–3). The 2009 guidelines for diastolic dysfunction included many parameters and was perceived as overly complex (4). In 2016, the guidelines were revised to simplify the measurement of LVDF, thereby enhancing the usefulness of the guidelines in routine practice (5, 6). It recommended two separate algorithms. For patients with maintained left ventricular ejection fraction (LVEF ≥ 50%) and unknown diastolic function, Algorithm A is primarily used to classify normal and abnormal diastolic function, while Algorithm B is designed to estimate LV filling pressure and grade diastolic function of patients with a reduced (<50%) or preserved LVEF and known or suspected diastolic dysfunction. However, if the patient's diagnosis is unknown or the LVEF is marginal (45–55%), there are problems in selecting an algorithm for LVDF evaluation. Therefore, there is a need for an LVDF assessment that can be easily applied to clinical practice by providers with different levels of expertise.

Recently, Oh et al. proposed a simplified and unified algorithm for LVDF assessment (7). This algorithm benefited by simplifying the assessment in clinical practice and avoiding problems with discordance and false calls of diastolic dysfunction to achieve high specificity. To date, the clinical impact of each individual LVDF marker is well-known, but the clinical significance of the sum of the abnormal diastolic function markers and the long-term clinical outcome are not well-known (8). This study aimed to investigate the usefulness of LVDF score in predicting clinical outcomes of patients with AMI.

## Materials and Methods

### Patient Population

All patients with AMI registered at Chonnam National University Hospital from 2011 to 2015 were included in the study. Of the initial 3,009 patients, 2,030 patients who underwent successful primary percutaneous intervention (PCI) and transthoracic echocardiography (TTE) were selected. Patients with moderate to severe mitral regurgitation (MR), mitral annular calcification, atrial fibrillation, those who did not undergo PCI, those who underwent suboptimal or failed PCI, those with no echocardiography findings, and those with insufficient TTE imaging or loss to follow-up were excluded (Supplementary Figure 1). AMI is defined as cardiomyocyte necrosis in a clinical setting consistent with acute myocardial ischemia (9). It was diagnosed by clinical presentation, serial changes on echocardiography suggesting infarction, and an increase in cardiac markers, preferably cardiac troponins, with at least one value above the 99th percentile of the upper reference limit. The study complies with the Declaration of Helsinki, and the local institutional review board (IRB) of the study center approved the study protocol (CNUH 05-49). Written informed consent was obtained from each study patient.

### Echocardiographic Data and Study Definition

A comprehensive transthoracic echocardiogram was obtained within 48 h of admission for all patients. All TTE measurements were recorded during routine clinical practice according to the current American Society of Echocardiography (ASE/EACVI) recommendations (10). Left ventricular systolic function was assessed by LVEF obtained using the biplane method of disk summation, from the apical 2- and 4-chamber views, according to the modified biplane Simpson's method. To calculate the wall motion score index (WMSI), the LV was divided into 16 segments. Each segment was assessed and scored based on its motion and systolic thickening (1 = normokinesia, 2 = hypokinesia, 3 = akinesia, 4 = dyskinesia). The WMSI was calculated as the sum of the individual segment scores divided by the number of segments (11). Left atrial (LA) volume was assessed using the modified biplane Simpson's method, from the apical 2- and 4-chamber views, at end-systole and indexed to body surface area. In cases in which the Simpson's method could not be used due to missing or poor quality apical views, LA volume index (LAVI) was calculated using the Cube method (12). Peak early diastolic tissue velocity (e') was measured from the septal aspects of the mitral annulus, while mitral inflow velocity was assessed using the pulsed-wave Doppler from the apical 4-chamber view (5). The right ventricular (RV) functional measures were tricuspid annulus systolic tissue Doppler velocity (s') and RV dysfunction, which was defined as s' < 10 cm/s. Peak tricuspid regurgitation (TR) velocity was measured, and pulmonary artery systolic pressure (PASP) was estimated as 4 × (peak TR velocity)^2^ + 5 (5).

Four LVDF parameters (septal e′ ≥ 7 cm/s, septal E/e′ ≤ 15, TR velocity ≤ 2.8 m/s, and LAVI ≤ 34 ml/m^2^) were used for LVDF scoring (7). The presence of each abnormal LVDF parameter was scored as 1, and the total LVDF score ranged from 0 to 4 (normal filling pressure: LVDF score 0–1, indeterminate: LVDF score 2, increased filling pressure: LVDF score 3–4).

### Clinical Data Collection

Demographic features and cardiovascular risk factors were obtained via patient interviews or review of medical records. During admission, findings of coronary angiography and detailed procedural characteristics of PCI, as well as data on discharge medications were collected. Patient treatment was performed according to current standard practice. After PCI, all patients were recommended to take aspirin indefinitely with clopidogrel or a potent P2Y12 inhibitor, such as prasugrel or ticagrelor, for at least 1 year.

### Clinical Outcomes

The incidence of mortality and hospitalization due to heart failure (HHF) in relation with the LVDF score over the 3-year study period were evaluated. All causes of death were considered cardiac unless an apparent non-cardiac cause was otherwise stated. Readmission for HF was defined as the patient showing signs and symptoms of HF upon admission and was treated with medications, including diuretic therapy (either intravenous diuretics or augmentation of oral diuretics), vasodilators, inotropic support, or ultrafiltration for HF during admission. All end points followed the definitions of the Academic Research Consortium (13).

### Statistical Analyses

Continuous variables are presented as means ± standard deviations or medians with interquartile ranges and compared using an unpaired *t*-test or Mann–Whitney rank sum test. Categorical variables are expressed as numbers with percentages and compared using Pearson's chi-square test or Fisher's exact test. Mortality and HHF were assessed using Kaplan–Meier curves according to the LVDF score. A multivariate Cox regression model was used for each of the above-mentioned cut-offs, with covariates that had *P* < 0.05 on univariate analysis or had predictive values [age ≥ 65 years, male sex, previous MI, estimated glomerular filtration rate (eGFR) ≤60%, LVEF, and cardiogenic shock, LV end-diastolic volume index, LV end-systolic volume index, LV geometry]. To compare the predictive abilities of LVDF scores and LVEF for mortality and HHF, receiver operating characteristic (ROC) curve analysis and DeLong's test were performed. In addition, comparisons of all-cause mortality between the LVDF score and LVEF according to the exploratory subgroups of interest were assessed using an ROC curve. For ROC curves, landmark analyses were used to compare LVDF scores and LVEF before and after 30 days of follow-up because 30 days following primary reperfusion is a critical period where the greatest degree of cardiac remodeling occurs (14).

All probability values were two-sided, and *p*-values <0.05 were considered statistically significant. All statistical analyses were performed using R Core Team (2015). R: A language and environment for statistical computing (version 3.6.0, R Foundation for Statistical Computing, Vienna, Austria. URL https://www.R-project.org/).

## Results

### Baseline Characteristics

**Figure 1:**
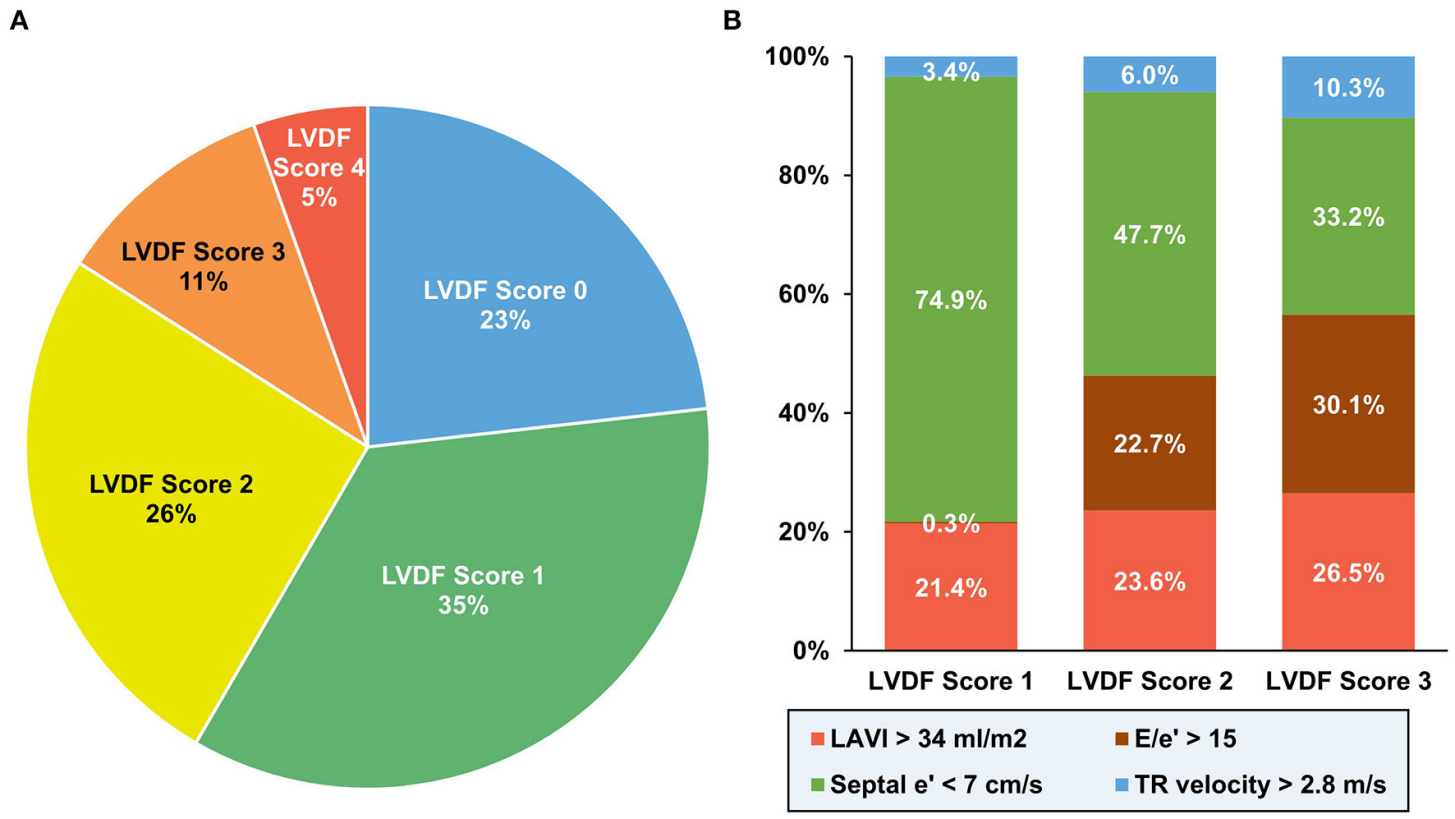
Distribution of diastolic function according to LVDF score. An LVDF score of 1 was the most prominent (35%) **(A)**, while an abnormal septal e' was the most common feature **(B)**. LAVI, left atrial volume index; LVDF, left ventricular diastolic function; TR, tricuspid regurgitation.

Of the 3,009 patients with AMI recruited for the study, 979 patients were excluded for following reasons: 39 with moderate to severe MR; 68 with mitral annular calcification; 194 with atrial fibrillation; 23 with failed or suboptimal PCI; 380 with no PCI; 93 with no TTE; 172 with insufficient TTE imaging; and 10 with follow-up loss (Supplementary Figure 1). The proportion of LVDF scores were as follows: 23.2% (score 0), 35.2% (score 1), 25.7% (score 2), 10.5% (score 3), and 5.4% (score 4) (Figure 1A). An LVDF score of 1 was the most prominent (35%), with an abnormal septal e' being the most common feature. E/e' > 15 showed an increasing pattern as the LVDF score increased (Figure 1B). The baseline demographics, final diagnosis, and risk factors were found to be significantly varied with the LVDF score (Table 1). A total of 2,030 patients with a mean age of 64.6 ± 12.6 years, including 1,471 males (72.5%), were included in this study. Co-morbidities such as hypertension and diabetes mellitus were found in 52.7 and 41.0% of patients, respectively. As the LVDF score increased, eGFR decreased while N-terminal pro-brain natriuretic peptide levels increased (*p* < 0.001). Second-generation drug-eluting stent was chosen as the most implanted intervention (85.1%), and the total number of stents was 1.4 ± 0.9. Most patients were receiving aspirin, a P2Y12 inhibitor, ACE inhibitor or ARB, beta-blocker, or a statin.

**Table 1:** Baseline Clinical and Procedural-related Characteristics.

	**Total** **(*N* = 2,030)**	**LVDF Score 0** **(*N* = 471)**	**LVDF Score 1** **(*N* = 714)**	**LVDF Score 2** **(*N* = 521)**	**LVDF Score 3** **(*N* = 214)**	**LVDF Score 4** **(*N* = 110)**	***P*-value**
**Demographics**							
Age, years	64.6 ± 12.6	54.9 ± 11.0	64.2 ± 11.6	69.5 ± 10.9	71.0 ± 11.1	72.8 ± 9.6	<0.001
Male	1,471 (72.5%)	418 (88.7%)	565 (79.1%)	315 (60.5%)	118 (55.1%)	55 (50.0%)	<0.001
Body mass index	24.1 ± 3.3	24.1 ± 3.0	24.3 ± 3.4	23.9 ± 3.4	23.9 ± 3.5	23.2 ± 3.5	0.016
**Comorbidities**							
Hypertension	1,070 (52.7%)	131 (27.8%)	372 (52.1%)	333 (63.9%)	154 (72.0%)	80 (72.7%)	<0.001
Diabetes mellitus	618 (30.4%)	96 (20.4%)	206 (28.9%)	169 (32.4%)	86 (40.2%)	61 (55.5%)	<0.001
Dyslipidemia	156 (7.7%)	43 (9.1%)	61 (8.5%)	23 (4.4%)	22 (10.3%)	7 (6.4%)	0.015
Previous history of MI	169 (8.3%)	21 (4.5%)	65 (9.1%)	35 (6.7%)	28 (13.1%)	20 (18.2%)	<0.001
Previous history of CVA	122 (6.0%)	13 (2.8%)	38 (5.3%)	42 (8.1%)	17 (7.9%)	12 (10.9%)	0.001
**Final diagnosis**							<0.001
STEMI	846 (41.7%)	224 (47.6%)	322 (45.1%)	191 (36.7%)	75 (35.0%)	34 (30.9%)	
NSTEMI	1,184 (58.3%)	247 (52.4%)	392 (54.9%)	330 (63.3%)	139 (65.0%)	76 (69.1%)	
**Examination and laboratory values**							
Systolic pressure	124.9 ± 22.3	123.5 ± 19.7	125.4 ± 22.2	124.3 ± 23.8	126.6 ± 24.4	127.7 ± 22.2	0.076
Diastolic pressure	78.4 ± 14.3	78.4 ± 13.4	78.7 ± 14.0	77.3 ± 15.1	79.1 ± 15.3	80.9 ± 14.7	0.476
Mean arterial pressure	93.8 ± 16.6	93.3 ± 15.1	94.2 ± 16.3	92.9 ± 17.6	94.8 ± 17.9	96.4 ± 16.8	0.221
Heart rate	78.9 ± 17.0	78.3 ± 16.1	77.6 ± 15.6	79.1 ± 18.4	81.5 ± 18.7	83.8 ± 18.0	<0.001
Killip class	1.4 ± 0.8	1.2 ± 0.6	1.3 ± 0.7	1.4 ± 0.8	1.6 ± 0.9	1.8 ± 0.9	<0.001
Cardiogenic shock	172 (8.5%)	24 (5.1%)	59 (8.3%)	53 (10.2%)	25 (11.7%)	11 (10.0%)	0.017
eGFR, ml/min/1.73 m^2^	96.2 ± 46.6	111.6 ± 40.9	99.4 ± 46.1	89.9 ± 49.3	83.4 ± 43.2	64.9 ± 38.5	<0.001
NT-proBNP, pg/ml	3474.2 ± 10216.1	860.1 ± 3230.2	2068.8 ± 5524.7	3051.8 ± 5816	6212.9 ± 8734.8	17068.7 ± 31162	<0.001
**Lesion profiles**							
Culprit vessel							0.286
Left main artery	52 (2.6%)	11 (2.3%)	17 (2.4%)	14 (2.7%)	5 (2.3%)	5 (4.5%)	
LAD artery	962 (47.4%)	226 (48.0%)	355 (49.7%)	228 (43.8%)	95 (44.4%)	58 (52.7%)	
Left circumflex artery	364 (17.9%)	75 (15.9%)	137 (19.2%)	97 (18.6%)	42 (19.6%)	13 (11.8%)	
Right coronary artery	652 (32.1%)	159 (33.8%)	205 (28.7%)	182 (34.9%)	72 (33.6%)	34 (30.9%)	
Three vessels disease	194 (9.6%)	27 (5.7%)	65 (9.1%)	60 (11.5%)	27 (12.6%)	15 (13.6%)	0.005
ACC/AHA B2/C lesion	1,893 (93.3%)	424 (90.0%)	668 (93.6%)	499 (95.8%)	197 (92.1%)	105 (95.5%)	0.006
Pre-PCI TIMI flow 0-1	1,035 (51.0%)	273 (58.0%)	360 (50.4%)	248 (47.6%)	103 (48.1%)	51 (46.4%)	0.01
**Procedural characteristics**							
Trans-radial approach	956 (47.1%)	222 (47.1%)	358 (50.1%)	239 (45.9%)	89 (41.6%)	48 (43.6%)	0.171
2nd generation DES	1,727 (85.1%)	418 (88.7%)	629 (88.1%)	433 (83.1%)	171 (79.9%)	76 (69.1%)	<0.001
Total number of stents	1.4 ± 0.9	1.4 ± 0.7	1.4 ± 0.8	1.5 ± 0.9	1.6 ± 1.0	1.4 ± 0.9	0.040
**Medication at discharge**							
Aspirin	2,029 (100.0%)	471 (100.0%)	714 (100.0%)	520 (99.8%)	214 (100.0%)	110 (100.0%)	0.575
P2Y12 inhibitor	2,028 (99.9%)	470 (99.8%)	714 (100.0%)	520 (99.8%)	214 (100.0%)	110 (100.0%)	0.716
Ticagrelor	460 (22.7%)	115 (24.4%)	165 (23.1%)	112 (21.5%)	45 (21.0%)	23 (20.9%)	
Prasugrel	516 (25.4%)	168 (35.7%)	206 (28.9%)	91 (17.5%)	34 (15.9%)	17 (15.5%)	
Clopidogrel	1,052 (51.8%)	187 (39.7%)	343 (48.0%)	317 (60.8%)	135 (63.1%)	70 (63.6%)	
ACE inhibitor or ARB	1,746 (86.0%)	408 (86.6%)	618 (86.6%)	450 (86.4%)	176 (82.2%)	94 (85.5%)	0.569
Beta-blocker	1,724 (84.9%)	411 (87.3%)	603 (84.5%)	432 (82.9%)	178 (83.2%)	100 (90.9%)	0.118
Statin	1,879 (92.6%)	447 (94.9%)	659 (92.3%)	482 (92.5%)	193 (90.2%)	98 (89.1%)	0.111

*Values are mean ± SD or n (%). NT-proBNP, N-terminal pro-brain natriuretic peptide; ACC/AHA, American College of Cardiology/American Heart Association; ACEI, angiotensin-converting enzyme inhibitor; ARB, angiotensin-II receptor blocker; CVA, cerebrovascular accident; DES, drug-eluting stent; eGFR, estimated glomerular filtration rate; LAD, left anterior descending artery; LVDF, left ventricular diastolic function; NSTEMI, non ST-segment elevation myocardial infarction; STEMI, ST-segment elevation myocardial infarction; TIMI, Thrombolysis in Myocardial Infarction*.

### Echocardiographic Characteristics

Based on the left ventricular mass index (LVMi) results and relative wall thickness, LV hypertrophy (LVH) was observed in 34.2% of patients (concentric: 12.9%, eccentric: 21.3%; Table 2). The prevalence of LVH increased as the LVDF score increased. The mean LVEF was 55.0 ± 11.3%. As the LVDF score increased, the LVEF decreased and WMSI increased. The higher the LVDF score of patients, the higher the LAVI and septal E/e' TR velocity but the lower the septal e'.

**Table 2:** Transthoracic Echocardiographic Characteristics.

**Variable**	**Total** **(*N* = 2,030)**	**LVDF score 0** **(*N* = 471)**	**LVDF score 1** **(*N* = 714)**	**LVDF score 2** **(*N* = 521)**	**LVDF score 3** **(*N* = 214)**	**LVDF score 4** **(*N* = 110)**	***P*-value**
**LV structure**							
LVEDVi (ml/m^2^)	72.0 ± 19.7	65.2 ± 14.2	67.9 ± 15.9	75.0 ± 20.0	84.7 ± 24.9	88.4 ± 23.6	<0.001
LVESVi (ml/m^2^)	31.5 ± 16.2	25.1 ± 9.5	28.5 ± 11.7	33.6 ± 17.5	42.7 ± 23.0	44.9 ± 20.5	<0.001
LVEDD (cm)	50.2 ± 5.8	49.0 ± 4.7	49.4 ± 5.3	50.5 ± 5.9	52.9 ± 7.0	53.5 ± 6.2	<0.001
LVESD (cm)	34.7 ± 6.8	32.5 ± 5.0	33.8 ± 5.7	35.2 ± 7.1	38.7 ± 8.8	39.5 ± 7.7	<0.001
Septal wall thickness (cm)	9.4 ± 1.8	9.2 ± 1.6	9.4 ± 1.7	9.4 ± 2.0	9.6 ± 2.0	9.6 ± 1.9	0.003
Posterior wall thickness (cm)	9.5 ± 1.5	9.4 ± 1.4	9.5 ± 1.5	9.5 ± 1.5	9.8 ± 1.8	9.9 ± 1.7	<0.001
LV mass (g)	171.9 ± 47.6	160.3 ± 39.8	167.4 ± 42.9	173.3 ± 49.4	193.9 ± 56.4	199.2 ± 53.6	<0.001
LV mass index (g/m^2^)	101.6 ± 26.8	91.1 ± 21.5	97.3 ± 23.0	104.6 ± 26.3	118.9 ± 31.0	124.5 ± 30.0	<0.001
RWT	0.39 ± 0.08	0.38 ± 0.07	0.39 ± 0.08	0.38 ± 0.07	0.38 ± 0.10	0.38 ± 0.09	0.056
**LV geometry**							<0.001
Normal	895 (48.2%)	268 (62.3%)	342 (52.4%)	198 (42.6%)	61 (31.0%)	26 (23.6%)	
Concentric remodeling	326 (17.6%)	100 (23.3%)	135 (20.7%)	66 (14.2%)	17 (8.6%)	8 (7.3%)	
Concentric hypertrophy	239 (12.9%)	24 (5.6%)	80 (12.3%)	73 (15.7%)	37 (18.8%)	25 (22.7%)	
Eccentric hypertrophy	395 (21.3%)	38 (8.8%)	96 (14.7%)	128 (27.5%)	82 (41.6%)	51 (46.4%)	
**LV systolic function**							
LVEF (%)	55.0 ± 11.3	59.2 ± 9.0	55.5 ± 10.2	54.3 ± 11.6	49.4 ± 12.8	47.8 ± 13.2	<0.001
≥50%	1434 (70.6%)	402 (85.4%)	520 (72.8%)	350 (67.2%)	112 (52.3%)	50 (45.5%)	
<50%	596 (29.4%)	69 (14.6%)	194 (27.2%)	171 (32.8%)	102 (47.7%)	60 (54.5%)	
TDI septal s' (cm/s)	6.7 ± 2.8	7.9 ± 1.8	7.0 ± 3.9	6.1 ± 1.6	5.6 ± 1.5	5.0 ± 1.7	<0.001
WMSI	1.4 ± 0.4	1.3 ± 0.3	1.4 ± 0.3	1.4 ± 0.4	1.6 ± 0.4	1.6 ± 0.4	<0.001
**LV diastolic function**							
E/A ratio	0.9 ± 2.1	1.0 ± 0.9	0.9 ± 3.3	0.8 ± 0.6	0.9 ± 0.6	1.4 ± 0.8	0.888
E wave (cm/s)	68.5 ± 21.1	70.0 ± 16.7	60.2 ± 17.6	66.9 ± 20.5	81.9 ± 20.3	98.4 ± 22.6	<0.001
A wave (cm/s)	80.2 ± 22.7	70.3 ± 18.4	78.8 ± 19.0	86.3 ± 22.6	92.4 ± 27.2	78.8 ± 31.3	<0.001
Deceleration time (ms)	205.0 ± 66.3	194.0 ± 54.2	211.4 ± 66.6	212.5 ± 67.3	202.6 ± 76.1	179.0 ± 73.6	0.831
TDI septal e' (cm/s)	5.8 ± 2.2	8.3 ± 2.0	5.7 ± 1.8	4.7 ± 1.3	4.3 ± 1.1	4.1 ± 1.1	<0.001
Septal E/e'	13.2 ± 6.8	8.6 ± 2.2	10.7 ± 2.4	15.0 ± 5.6	20.4 ± 8.7	25.4 ± 9.6	<0.001
**LA size and function**							
LA diameter (mm)	38.5 ± 5.8	35.3 ± 3.5	36.8 ± 5.2	39.7 ± 5.6	43.1 ± 5.0	46.4 ± 4.4	<0.001
LAVI (cm^2^/m^2^)	32.2 ± 14.7	23.7 ± 6.3	27.8 ± 11.6	35.1 ± 14.0	44.5 ± 14.7	54.5 ± 16.0	<0.001
TDI septal a' (cm/s)	9.0 ± 2.3	9.9 ± 2.4	9.3 ± 2.1	8.6 ± 2.0	7.9 ± 2.0	6.7 ± 2.1	<0.001
**RV function**							
TDI RV s' (cm/s)	12.4 ± 2.9	12.4 ± 2.4	12.3 ± 3.0	12.4 ± 3.1	12.5 ± 2.9	12.7 ± 3.3	0.445
**Pulmonary pressure**							
TR velocity (m/s)	2.1 ± 0.7	1.8 ± 0.7	1.9 ± 0.6	2.1 ± 0.7	2.5 ± 0.6	3.2 ± 0.3	<0.001
PASP (mmHg)	24.6 ± 11.7	20.2 ± 8.9	21.8 ± 9.0	24.9 ± 11.0	31.7 ± 11.9	46.8 ± 8.5	<0.001

*Values are expressed as mean ± standard deviation or number (%)*.

*A wave, peak late diastolic velocity of mitral inflow; AV, aortic valve; A' wave, peak late diastolic velocity of mitral septal annulus; DT, deceleration time of mitral inflow; E wave, peak early diastolic velocity of mitral inflow; E' wave, peak early diastolic velocity of mitral septal annulus; LAD, left atrium diameter; LVEDVi, left ventricular end-diastolic volume index; LVEDD, left ventricular end-diastolic diameter; LVEF, left ventricular ejection fraction; LVESVi, left ventricular end-systolic volume index; LVESD, left ventricular end-systolic diameter; MR, mitral regurgitation; PG, pressure gradient; PASP, pulmonary artery systolic pressure; RWT: relative wall thickness; s': peak systolic velocity of mitral septal annulus; TDI, tissue doppler imaging; TRV, tricuspid regurgitation velocity; WMSI, wall motion score index*.

### Clinical Outcome

**Figure 2:**
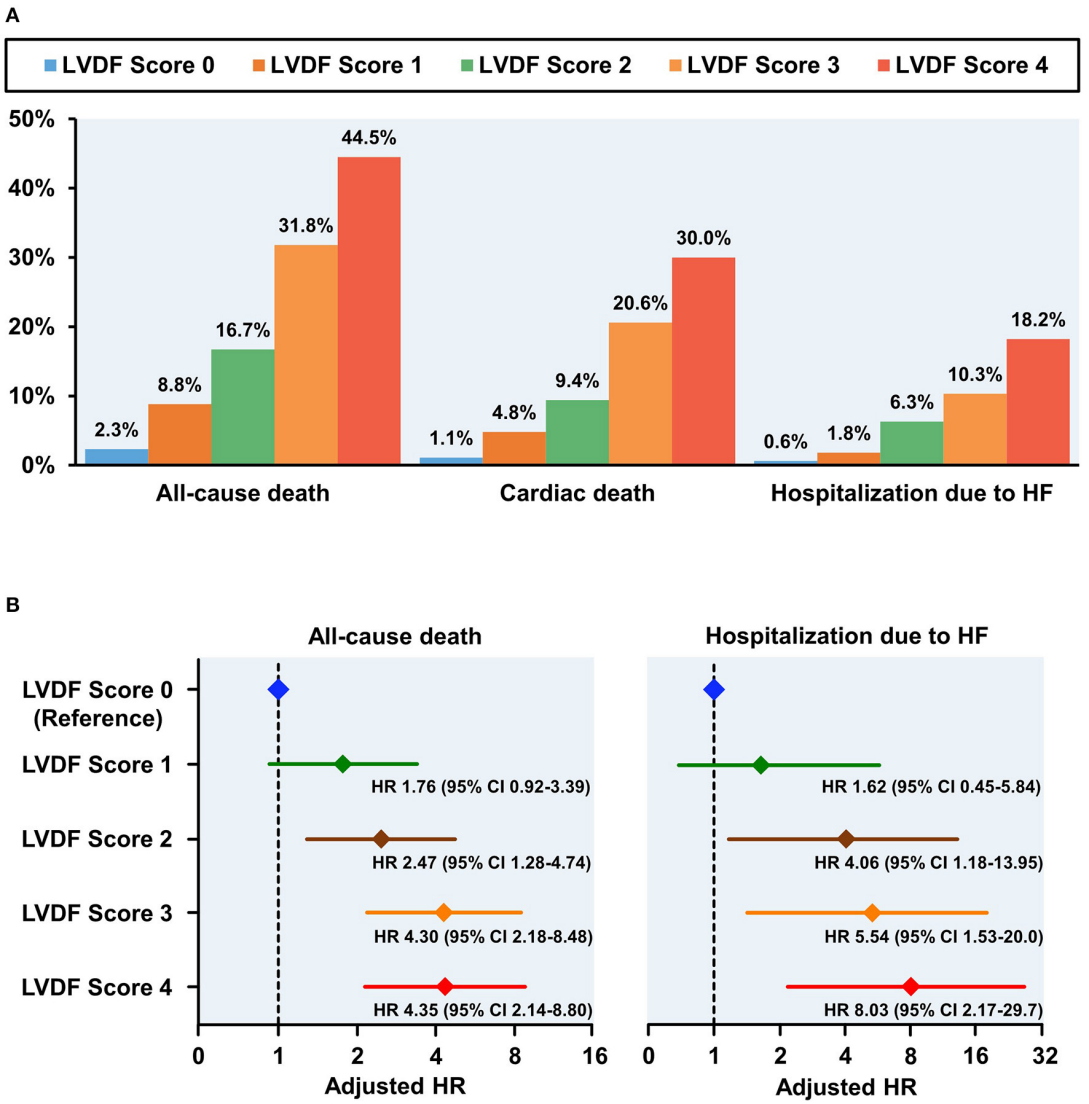
Clinical outcomes according to LVDF scores **(A)** and adjusted HR plot for all-cause mortality and hospitalization due to HF **(B)**. All-cause mortality, cardiac death, and heart failure (HF) rehospitalization rates increased in a stepwise fashion from 2.3% (LVDF score 0) to 44.5% (LVEF score 4) (*p* < 0.001). Higher LVDF scores had incrementally higher adjusted HRs for all-cause mortality and hospitalization due to HF. CI, confidence interval; HF, heart failure; HR, hazard ratio; LVDF, left ventricular diastolic function.

**Figure 3:**
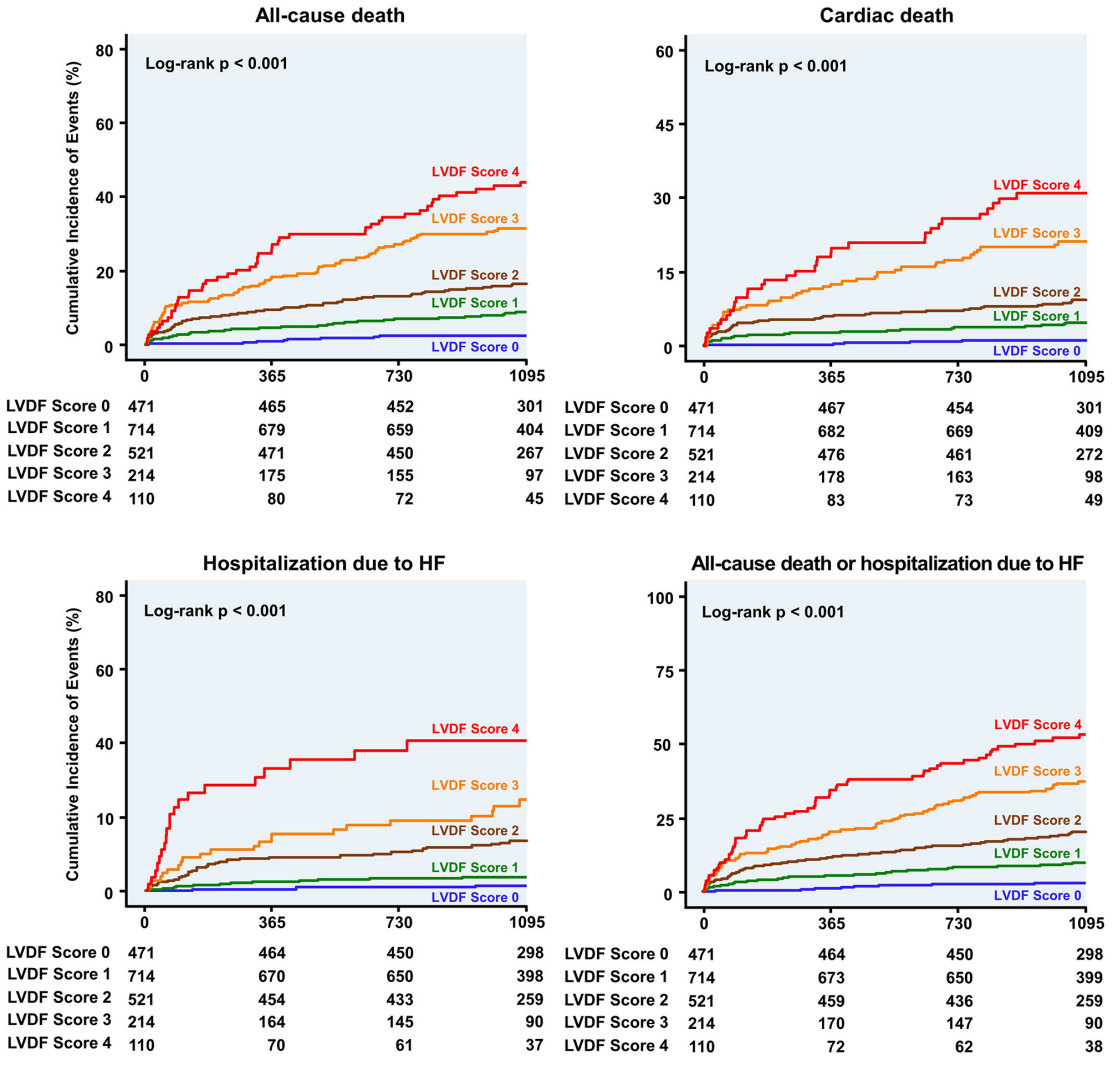
Kaplan–Meier curves for clinical outcome according to the LVDF score. Patients with higher LVDF scores had a higher rate of all-cause mortality, cardiac death, or hospitalization due to HF than patients with lower LVDF scores (log-rank *p* < 0.001). HF, heart failure; HR, hazard ratio; LVDF, left ventricular diastolic function.

During a median follow-up period of 1,099 (interquartile range: 1,063–1,124) days, 278 patients (13.7%) died and 91 patients (4.5%) were readmitted for HF. All-cause mortality and HHF significantly increased as the LVDF score increased (all-cause mortality–LVDF score 0: 2.3%, score 1: 8.8%, score 2: 16.7%, score 3: 31.8%, and score 4: 44.5%, *p* < 0.001; HHF–LVDF score 0: 0.6%, score 1: 1.8%, score 2: 6.3%, score 3: 10.3%, and score 4: 18.2%, *p* < 0.001; Figure 2A). In multivariate analysis, a higher LVDF score was associated with significantly higher adjusted hazard ratios (HR) for all-cause mortality and HHF (Figure 2B). Kaplan-Meier survival analysis revealed that patients with higher LVDF scores had a higher rate of all-cause mortality, cardiac death, and HHF than those with lower LVDF scores (log-rank *p* < 0.001; Figure 3). In various subgroups, the LVDF scores stratification had incrementally higher adjusted hazard ratio (HR) for all-cause mortality (Supplementary Figure 2).

LVDF score 2–4 and LVEF < 40% were independent predictors of all-cause death, and LVDF score 2–4, LVEF 40–50% and <40% were independent predictors of HHF (Table 3). In addition, HR values for all-cause death and HHF were higher in LVDF score 4 than in LVEF < 40% {all-cause death—LVDF score 4: HR 4.346 [95% confidence interval (CI) 1.468–2.933], *p* < 0.001) vs. LVEF < 40%: HR 2.075 (95% CI 1.468–2.933), *p* < 0.001; HHF–LVDF score 4: HR 8.031 (95% CI 2.169–29.73), *p* = 0.002 vs. LVEF < 40%: HR 3.206 (95% CI 1.758–5.847), *p* = 0.001}.

**Table 3:** Independent Predictors for All-cause Death And Hospitalization Due to HF.

	**HR**	**95% CI**	***P*-value**
**All-cause death**
LVDF score 1 (LVDF Score 0 as a reference)	1.761	0.915 – 3.387	0.090
LVDF score 2 (LVDF Score 0 as a reference)	2.468	1.284 – 4.743	0.007
LVDF score 3 (LVDF Score 0 as a reference)	4.299	2.180 – 8.475	<0.001
LVDF score 4 (LVDF Score 0 as a reference)	4.346	2.144 – 8.804	<0.001
LVEF 40-50% (LVEF ≥ 50% as a reference)	1.325	0.963 – 1.823	0.083
LVEF < 40% (LVEF ≥ 50% as a reference)	2.075	1.468 – 2.933	<0.001
Age > 65 years	5.338	3.518 – 8.100	<0.001
Male	1.190	0.919 – 1.542	0.187
Previous history of MI	1.819	1.312 – 2.522	0.001
eGFR < 60	2.241	1.752 – 2.868	<0.001
Cardiogenic shock	2.739	2.038 – 3.684	<0.001
LVEDVi ≥ 31.5 ml/m^2^	1.082	0.798 – 1.467	0.611
LV mass index ≥ 101 ml/m^2^	0.991	0.724 – 1.358	0.957
Abnormal LV geometry	1.263	0.925 – 1.726	0.142
**Hospitalization due to HF**
LVDF score 1 (LVDF Score 0 as a reference)	1.624	0.451 – 5.838	0.458
LVDF score 2 (LVDF Score 0 as a reference)	4.064	1.183 – 13.95	0.026
LVDF score 3 (LVDF Score 0 as a reference)	5.535	1.531 – 20.00	0.009
LVDF score 4 (LVDF Score 0 as a reference)	8.031	2.169 – 29.73	0.002
LVEF 40-50% (LVEF ≥ 50% as a reference)	1.773	1.018 – 3.086	0.043
LVEF < 40% (LVEF ≥ 50% as a reference)	3.206	1.758 – 5.847	0.001
Age > 65 years	2.223	1.233 – 4.008	0.008
Male	0.862	0.551 – 1.348	0.515
Previous history of MI	1.268	0.692 – 2.321	0.442
eGFR < 60	2.798	1.826 – 4.289	<0.001
Cardiogenic shock	1.362	0.740 – 2.506	0.320
LVEDVi ≥ 31.5 ml/m^2^	1.219	0.702 – 2.117	0.482
LV mass index ≥ 101 ml/m^2^	0.830	0.468 – 1.470	0.523
Abnormal LV geometry	1.469	0.832 – 2.596	0.185

*Hazard ratios and their 95% confidence intervals were calculated by multivariable Cox regression analysis*.

*Abbreviations are as in Tables 1, 2*.

### Prediction of Clinical Outcome: LVDF Score vs. LVEF

**Figure 4:**
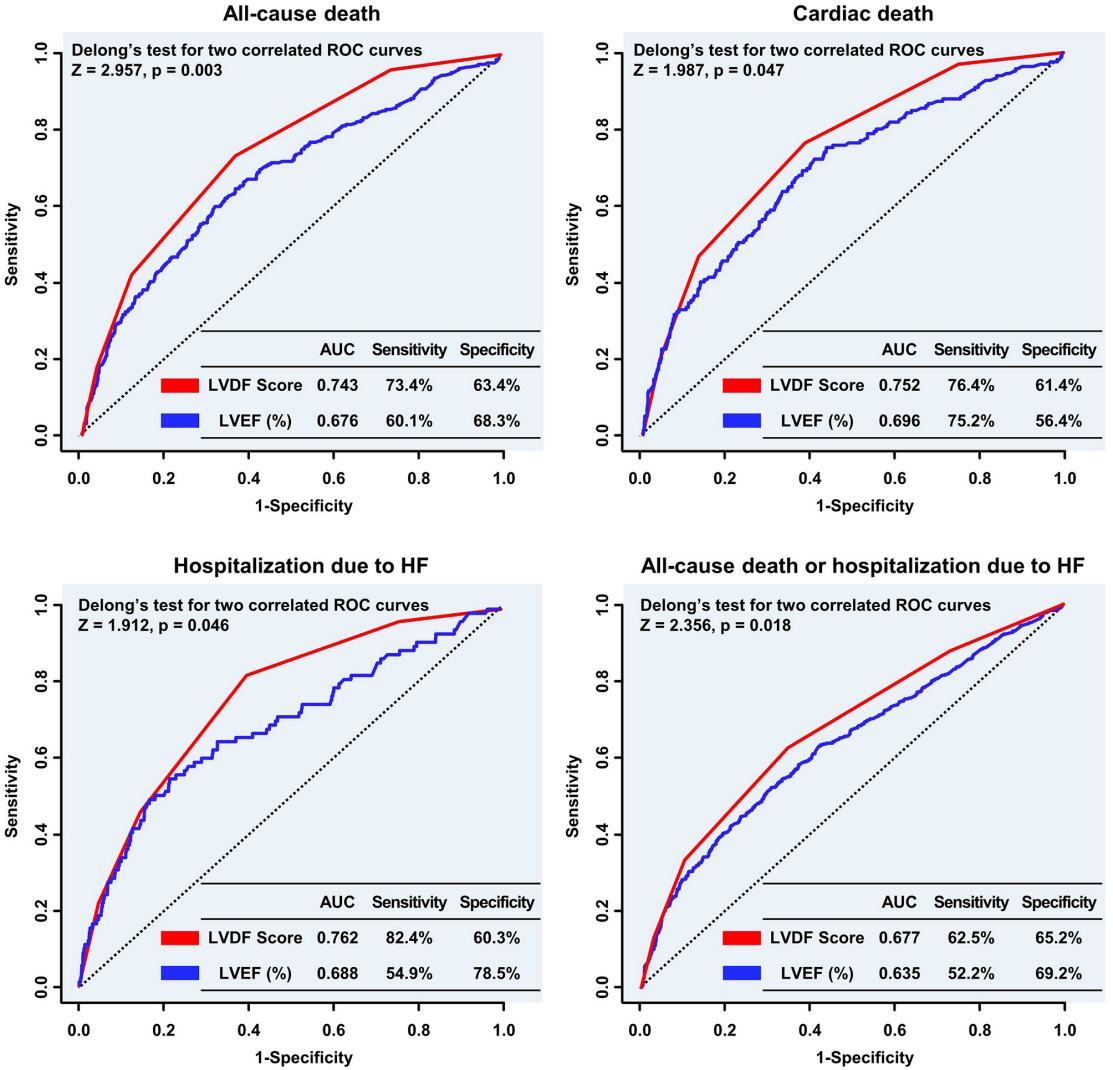
Comparison of the predictive performance of the LVDF score and LVEF for clinical outcomes. The LVDF score performed significantly better in predicting all-cause mortality, cardiac death, and hospitalization due to HF compared with LVEF. AUC, area under the receiver operating characteristic curve; HF, heart failure; LVDF, left ventricular diastolic function; LVEF, left ventricular ejection fraction; ROC, receiver operating characteristic.

The ROC curve showed that the LVDF score was significantly better at predicting all-cause mortality and readmission for recurrent HF than LVEF (area under the ROC curve [AUC]: 0.743 vs. 0.676, Delong's test *p* = 0.003; AUC: 0.762 vs. 0.688, Delong's test *p* = 0.046; respectively; Figure 4).

Subgroup analysis showed that the LVDF score performed significantly better than LVEF in patients with ST-segment elevation myocardial infarction (STEMI), LVEF ≥ 50%, Killip class < 3, abnormal LV geometry (LV remodeling or LVH), and non-right coronary artery (RCA) target vessel (Supplementary Figure 3). Comparison of the predictive performance of the individual LVDF parameters and LVEF showed that E/e' ratio was the best predictor of all-cause death and HHF (Supplementary Figure 4).

**Figure 5:**
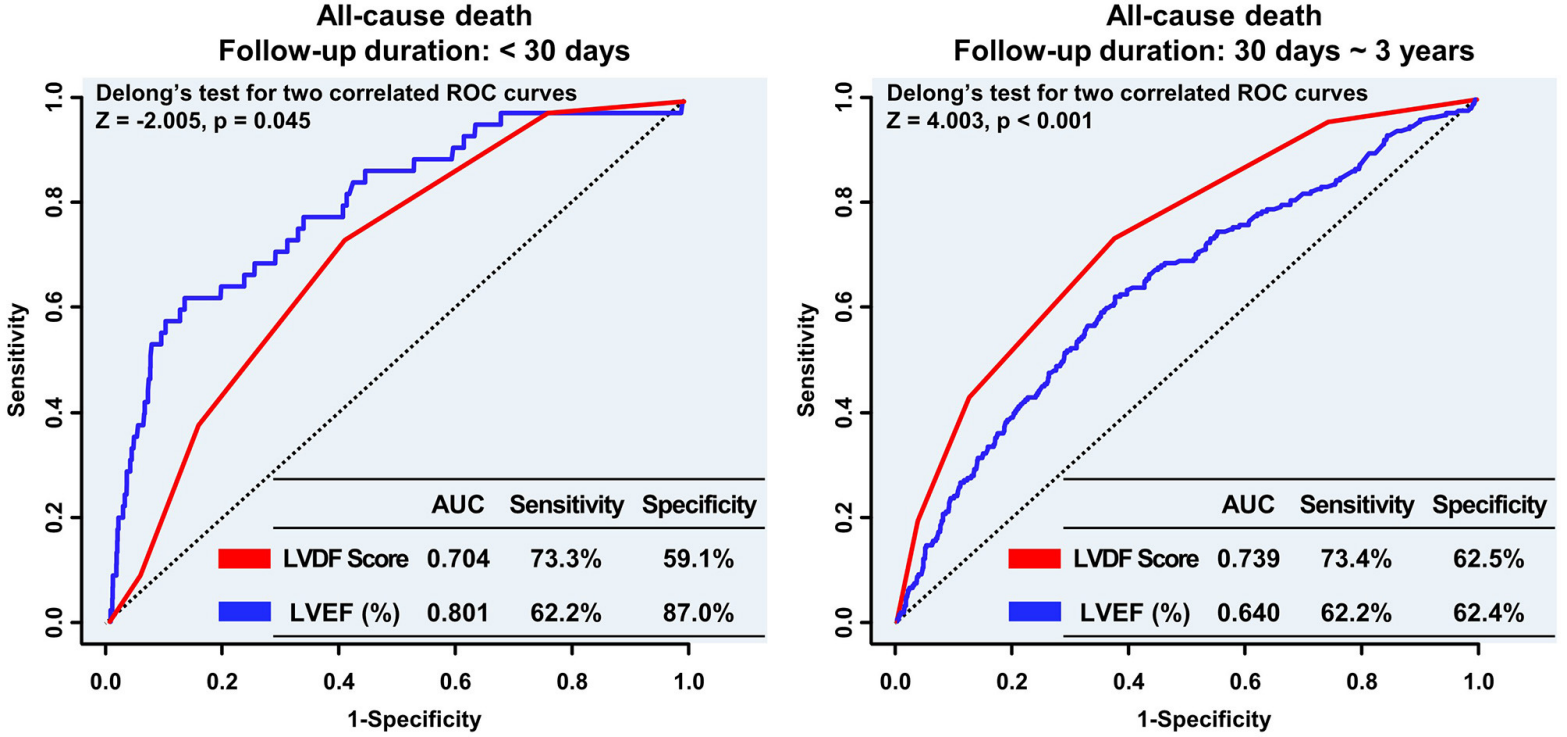
Landmark analyses of the ROC curve to compare the LVDF score and LVEF before and after the 30-day follow-up period. In the landmark analysis, LVEF was the strongest predictor of all-cause mortality during the short-term follow up; however, the LVDF score was a better predictor of mortality than LVEF during the long-term follow-up. AUC, area under the receiver operating characteristic curve; LVDF, left ventricular diastolic function; LVEF, left ventricular ejection fraction; ROC, receiver operating characteristic.

In landmark analysis, LVEF was the most predictive parameter for all-cause mortality within the first 30 days of follow-up (AUC: 0.801 vs. 0.704, *p* = 0.045), but between 30 days and 3 years of follow-up, the LVDF score was the better predictor (AUC: 0.739 vs. 0.640, *p* < 0.001) (Figure 5).

## Discussion

This study examined the construct validity of the unified LVDF algorithm, by demonstrating the ability of a simplified LVDF score to outperform LV systolic function in predicting long-term clinical outcomes in 2,030 patients with AMI. Patients with high LVDF scores had a significantly higher risk of all-cause mortality or readmission for recurrent HF than patients with low LVDF scores, which was consistently observed even after adjusting for baseline differences. The LVDF score performed significantly better in predicting all-cause mortality and readmission for recurrent HF compared with LVEF. Subgroup analysis showed that LVDF scores performed significantly better than LVEF in patients with STEMI, LVEF ≥ 50%, Killip class < 3, abnormal LV geometry (LV remodeling or LVH), and non-RCA target vessels. In landmark analysis, LVDF scores were better in predicting all-cause mortality than LVEF in the long-term follow-up (30 days ~ 3 years).

### Predicting Clinical Outcome Using the LVDF Score in Patients With Acute Myocardial Infarction

Previous studies have shown that in the 2016 ASE/EACVI guidelines, assessment of diastolic function was a strong independent predictor of outcomes for MI (15, 16). However, these studies excluded indeterminate variables and shock groups, had relatively smaller study subjects, and included limitations that were difficult to apply in a clinical setting. Contrastingly, the present study included patients with AMI with cardiogenic shock and indeterminate variables and found that the prognosis worsened as the LVDF score increased. Additionally, in the distribution of diastolic function, the most common in LVDF score 1 was septal e' < 7 cm/s. However, in LVDF score 2, the E/e' > 15 ratio increases, and thereafter, in LVDF score 3, the TR velocity > 2.8 m/s ratio increases. And among individual LVDF parameters, E/e' ratio and TR velocity were the best predictors of all-cause death and HHF. Therefore, clinical outcomes can be predicted simply by evaluating the LVDF scoring system, which is expected to be helpful in routine clinical practice for patients with AMI.

### Comparison of LVDF Score to LVEF for Mortality Prediction

Prognosis of LV systolic dysfunction after AMI has been a major research focus for several decades (3). The insights from these studies have led to several therapeutic interventions that have improved outcomes. In addition to depressed systolic function, clinical and radiological evidence of HF is a consistent and powerful predictor of outcomes in patients with AMI (17). However, there have been no studies comparing mortality between the two predictors, namely LV systolic function and current LVDF guidelines, in patients with AMI. In the present study, the LVDF score was found to be superior to LVEF in predicting mortality, especially in patients with AMI with STEMI, preserved LVEF (≥50%), hemodynamic stable state (Killip class <3), abnormal LV geometry, and non-RCA target vessels. LVEF is a strong predictor for clinical outcomes; however, since each LVDF parameter, including septal e', E/e', TR velocity, and LAVI, is known as a strong independent prognostic factor in HF and other diseases (18–21), the intersection of these four can be judged as a more powerful predictor for mortality. Especially, abnormal LV geometry (increased wall thickness and/or reduced end diastolic volume), which is a confounder for LVEF, makes it possible for LVEF to be unaltered despite significantly reduced LV function (22). In this study, as a result of analyzing the LV of patients with AMI, the normal LV geometry was less than half and the total LV mass index was 101.6 ± 26.8 g/m^2^, which was thicker compared with the LV wall of the normal population (69.9 ± 8.9 g/m^2^) (23); thus, LVDF is considered to be a better predictor than LVEF.

### The Importance of Evaluating LV Diastolic Function

There are prognostic reasons why LVDF evaluation is clinically important. From a diagnostic point of view, elevated LV filling pressure is an important cause of HF in patients with AMI (24). There are several studies focusing on optimal non-invasive assessment of left ventricular filling pressures that compared natriuretic peptide levels with Doppler against mean wedge pressure. Studies have shown that Doppler had a stronger correlation with mean wedge pressure, and the E/e' ratio tracked with changes in mean wedge pressure, whereas B-type natriuretic peptide levels did not (25). Similar results were seen in patients with ambulatory HF in which the E/e' ratio successfully tracked with changes in LA pressure (26). In this study, there was no significant difference in MAP in all groups (*p* = 0.221), but the LVDF score increased with the E/e' ratio (*p* < 0.001), leading to a decrease in coronary perfusion pressure, which is thought to be the cause of increased mortality and readmission for recurrent HF in the long term.

Interestingly, LVEF was superior to the LVDF score for predicting all-cause mortality during the short-term follow-up period (<30 days), but the LVDF score was superior to LVEF during the long-term follow-up (30 days ~ 3 years). As a consequence of AMI, the measurement of changes in LV size, shape, and the thickness of both infarcted and non-infarcted segments of the ventricle, collectively referred to as ventricular remodeling, is important in evaluating ventricular function and prognosis (27); however, several studies have shown that measurement of lesion size and left ventricular systolic function (28, 29) or alterations in post-infarction left ventricular remodeling (30) do not explain why patients with AMI have an increased tendency to develop long-term adverse outcomes. Therefore, in the acute stage, assessing prognosis based on LVEF is reasonable, and it is desirable to assess the prognosis using the LVDF score for patients undergoing long-term follow-up.

### Study Limitations

This study had several limitations. First, despite its large sample size and granular data, this study had the potential for unmeasured confounders and lack of some data. Second, echocardiography-based estimates of hemodynamic measurements, such as the E/e′ ratio, were used to measure LV filling pressures and TR velocity for pulmonary artery pressures, which are indirect measures. However, these correlate well with invasive measurements (31), and in clinical practice, diastolic function is evaluated mainly using echocardiography. Third, the 2016 ASE/EACVI guidelines recommended using the average of the lateral and septal velocities to measure LVDF, since these values are significantly different in certain situations such as left bundle branch block, regional wall motion abnormality, or significant right ventricular dysfunction, but only the septal e' velocity was used. However, there is no evidence that the average e' velocity provides a more reliable assessment for diastolic function (7). Moreover, septal E/e' was found to be associated with a poor outcome in TOPCAT trial (32), whereas lateral E/e' did not differ between patients with heart failure with preserved ejection fraction who were and were not hospitalized in I-Preserve (33). In the present study, comparison of the predictive performance of the individual LVDF parameters showed that E/e' ratio was the best predictor of all-cause death and hospitalization due to HF (Supplementary Figure 4). Subgroup analysis was performed for factors that could affect septal e' (WMSI ≥ 2, left bundle branch block or TDI RV s' < 9.5 cm/s) (Supplementary Figure 5). Therefore, consistent results were obtained.

## Conclusion

The present study demonstrated that the LVDF score is a significant predictor of mortality and HHF in patients with AMI. The LVDF score can be useful in the risk stratification of patients with AMI; thus, careful monitoring and management should be provided to patients with AMI with higher LVDF scores.

## Data Availability Statement

The raw data supporting the conclusions of this article will be made available by the authors, without undue reservation.

## Ethics Statement

The studies involving human participants were reviewed and approved by the study complies with the Declaration of Helsinki, and the Chonnam national university hospital institutional review board of the study center approved the study protocol (CNUH 05-49). The patients/participants provided their written informed consent to participate in this study.

## Author Contributions

SB performed the statistical analysis. SB and HY drafted the manuscript. KK, HK, HP, JC, MK, YK, YA, JC, and MJ reviewed/edited the manuscript and contributed to the interpretation of data. KK conceptualized the overall study design and supervised all aspects of the study and revised the manuscript critically. All authors have read and approved the manuscript.

## Funding

This study was supported by a grant of Chonnam National University Hospital Biomedical Research Institute (BCRI19039).

## Conflict of Interest

The authors declare that the research was conducted in the absence of any commercial or financial relationships that could be construed as a potential conflict of interest.

## Publisher's Note

All claims expressed in this article are solely those of the authors and do not necessarily represent those of their affiliated organizations, or those of the publisher, the editors and the reviewers. Any product that may be evaluated in this article, or claim that may be made by its manufacturer, is not guaranteed or endorsed by the publisher.

## Abbreviations

AMI: acute myocardial infarction
AUC: area under the receiver operating characteristic curve
eGFR: estimated glomerular filtration rate
HF: heart failure
LAVI: left atrial volume index
LV: left ventricular
LVDF: left ventricular diastolic function
LVEF: left ventricular ejection fraction
LVH: left ventricular hypertrophy
PCI: percutaneous coronary intervention
RCA: right coronary artery
ROC: receiver operating characteristic
RV: right ventricular
STEMI: ST-segment elevation myocardial infarction
TR: tricuspid regurgitation
TTE: transthoracic echocardiography
WMSI: wall motion score index.
