# Supplementary material for: Usefulness of Diastolic Function Score as a Predictor of Long-Term Prognosis in Patients With Acute Myocardial Infarction
Source: Front Cardiovasc Med. 2021 Sep 10;8:730872. doi: 10.3389/fcvm.2021.730872 (PMC8460859; doi:10.3389/fcvm.2021.730872)
Supplement: Supplementary file 1 [file Data_Sheet_1.pdf]

Supplementary Online Content

Supplementary Table 1. Independent Predictors for Clinical Outcomes

Supplementary Figure 1. Study flow.

Supplementary Figure 2. Exploratory subgroup analysis for all-cause death

Supplementary Figure 3. Comparison of the predictive performance of the LVDF score and LVEF for mortality in the subgroups

Supplementary Figure 4. Comparison of the predictive performance of the individual LVDF parameters and LVEF for all-cause death and hospitalization due to HF

Supplementary Figure 5. Subgroup analysis for factors that could affect septal e'

Supplementary Table 1. Independent Predictors for Clinical Outcomes

	HR	95% CI	P value
**Cardiac death**			
LVDF score 1 (LVDF Score 0 as a reference)	1.877	0.722 – 4.875	0.196
LVDF score 2 (LVDF Score 0 as a reference)	2.606	1.008 – 6.731	0.047
LVDF score 3 (LVDF Score 0 as a reference)	4.867	1.844 – 12.83	0.001
LVDF score 4 (LVDF Score 0 as a reference)	4.812	1.771 – 13.07	0.002
LVEF 40-50% (LVEF ≥ 50% as a reference)	1.115	0.723 – 1.717	0.623
LVEF < 40% (LVEF ≥ 50% as a reference)	2.264	1.472 – 3.482	0.001
Age > 65 years	6.258	3.474 – 11.27	<0.001
Male	0.976	0.699 – 1.361	0.884
Previous history of MI	2.621	1.785 – 3.849	<0.001
eGFR < 60	2.177	1.584 – 2.992	<0.001
Cardiogenic shock	3.858	2.697 – 5.519	<0.001
LVEDVi ≥ 31.5 ml/m^2	1.132	0.757 – 1.694	0.545
LV mass index ≥ 101 ml/m^2	1.204	0.788 – 1.840	0.392
Abnormal LV geometry	1.068	0.703 – 1.622	0.757
**Non-cardiac death**			
LVDF score 1 (LVDF Score 0 as a reference)	1.118	0.515 – 2.425	0.779
LVDF score 2 (LVDF Score 0 as a reference)	1.523	0.698 – 3.322	0.291
LVDF score 3 (LVDF Score 0 as a reference)	2.365	1.012 – 5.525	0.046
LVDF score 4 (LVDF Score 0 as a reference)	2.390	1.059 – 5.949	0.031
LVEF 40-50% (LVEF ≥ 50% as a reference)	1.683	1.057 – 2.679	0.028
LVEF < 40% (LVEF ≥ 50% as a reference)	1.651	0.915 – 2.977	0.096
Age > 65 years	4.521	2.516 – 8.122	<0.001
Male	1.516	1.004 – 2.288	0.047
Previous history of MI	0.875	0.453 – 1.690	0.689
eGFR < 60	2.579	1.754 – 3.790	<0.001
Cardiogenic shock	1.527	0.886 – 2.631	0.128
LVEDVi ≥ 31.5 ml/m^2	1.002	0.633 – 1.585	0.994
LV mass index ≥ 101 ml/m^2	0.857	0.538 – 1.367	0.518
Abnormal LV geometry	1.499	0.941 – 2.389	0.089
**All-cause death or HF rehospitalization**			
LVDF score 1 (LVDF Score 0 as a reference)	1.683	0.937 – 3.024	0.081
LVDF score 2 (LVDF Score 0 as a reference)	2.532	1.412 – 4.536	0.002
LVDF score 3 (LVDF Score 0 as a reference)	4.207	2.288 – 7.733	<0.001
LVDF score 4 (LVDF Score 0 as a reference)	4.744	2.517 – 8.942	<0.001
LVEF 40-50% (LVEF ≥ 50% as a reference)	1.394	1.041 – 1.865	0.003
LVEF < 40% (LVEF ≥ 50% as a reference)	2.288	1.671 – 3.133	<0.001
Age > 65 years	4.193	2.944 – 5.971	<0.001
Male	1.148	0.905 – 1.455	0.256

Previous history of MI	1.67	1.233 – 2.264	0.001
eGFR < 60	2.396	1.911 – 3.002	<0.001
Cardiogenic shock	2.34	1.769 – 3.095	<0.001
LVEDVi ≥ 31.5 ml/m^2	1.159	0.876 – 1.532	0.302
LV mass index ≥ 101 ml/m^2	0.932	0.697 – 1.246	0.635
Abnormal LV geometry	1.296	0.974 – 1.723	0.075

Hazard ratios and their 95% confidence intervals were calculated by multivariable Cox regression analysis.

Abbreviations are as in Tables 1 and 2.

Supplementary Figure 1. Study flow.

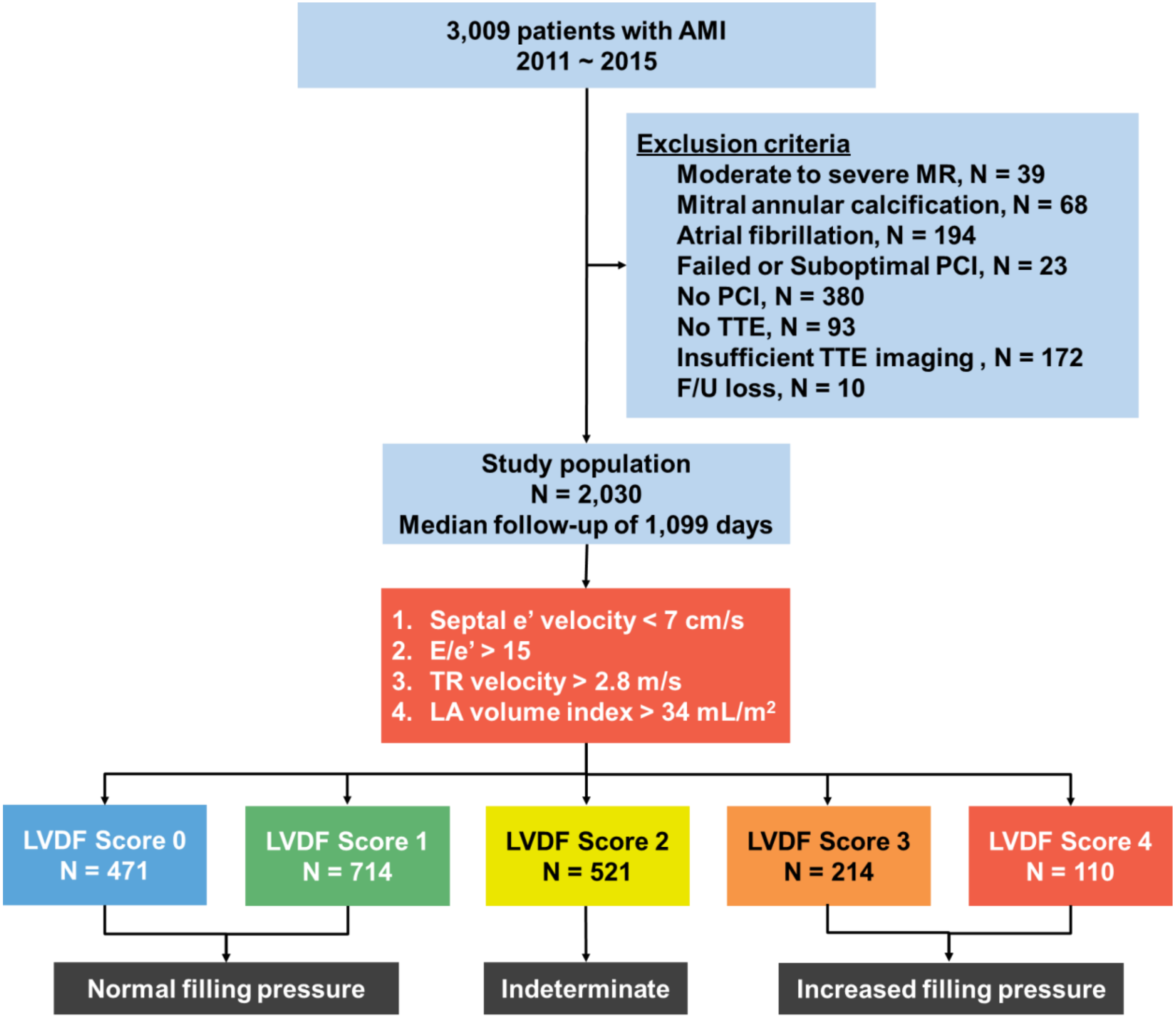

AMI, acute myocardial infarction; LA, left atrium; LVDF, left ventricular diastolic function; MR, mitral regurgitation; PCI, primary percutaneous intervention; TR, tricuspid regurgitation; TTE, transthoracic echocardiography.

Supplementary Figure 2. Exploratory subgroup analysis for all-cause death

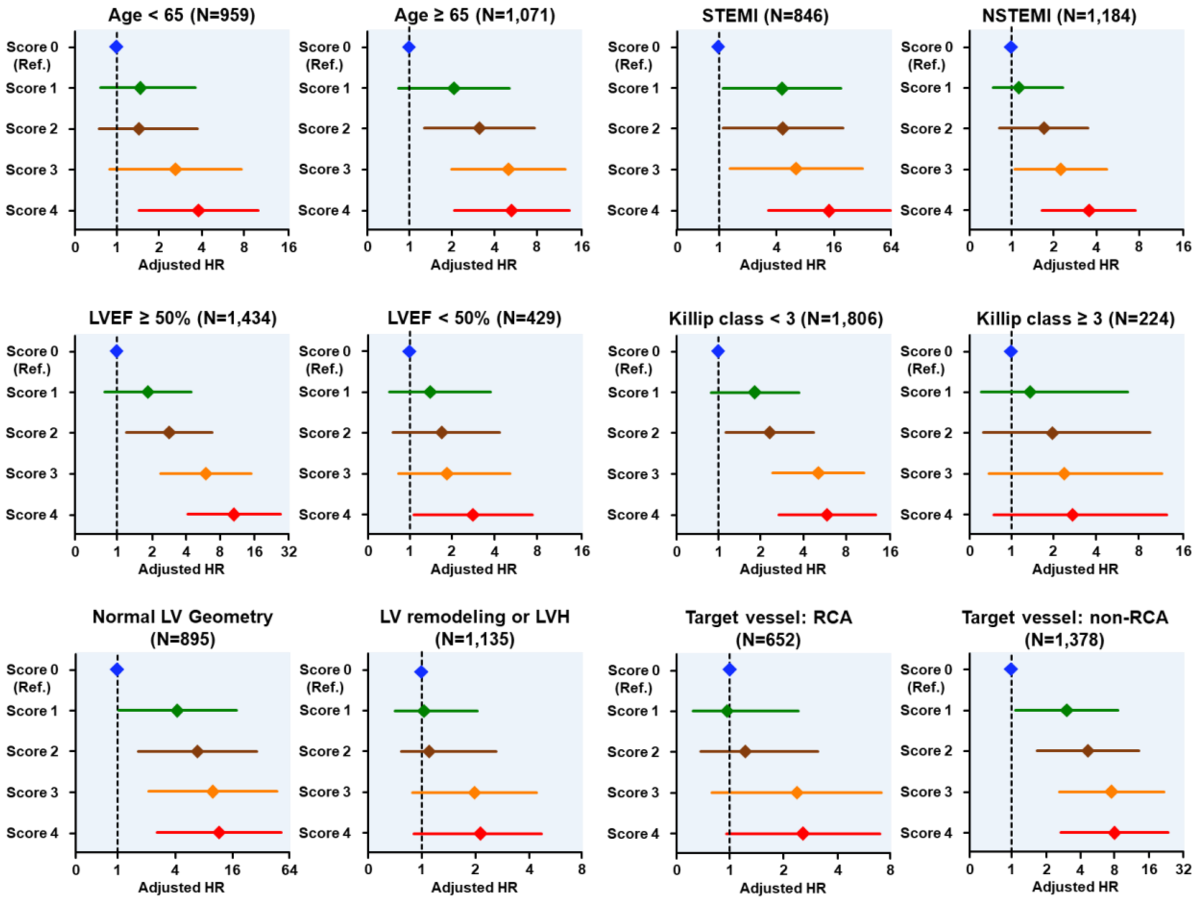

LVEF, left ventricular ejection fraction; LVH, left ventricular hypertrophy; NSTEMI, non ST-segment elevation myocardial infarction; STEMI, ST-segment elevation myocardial infarction; RCA, right coronary artery.

Supplementary Figure 3. Comparison of the predictive performance of the LVDF score and LVEF for mortality in the subgroups

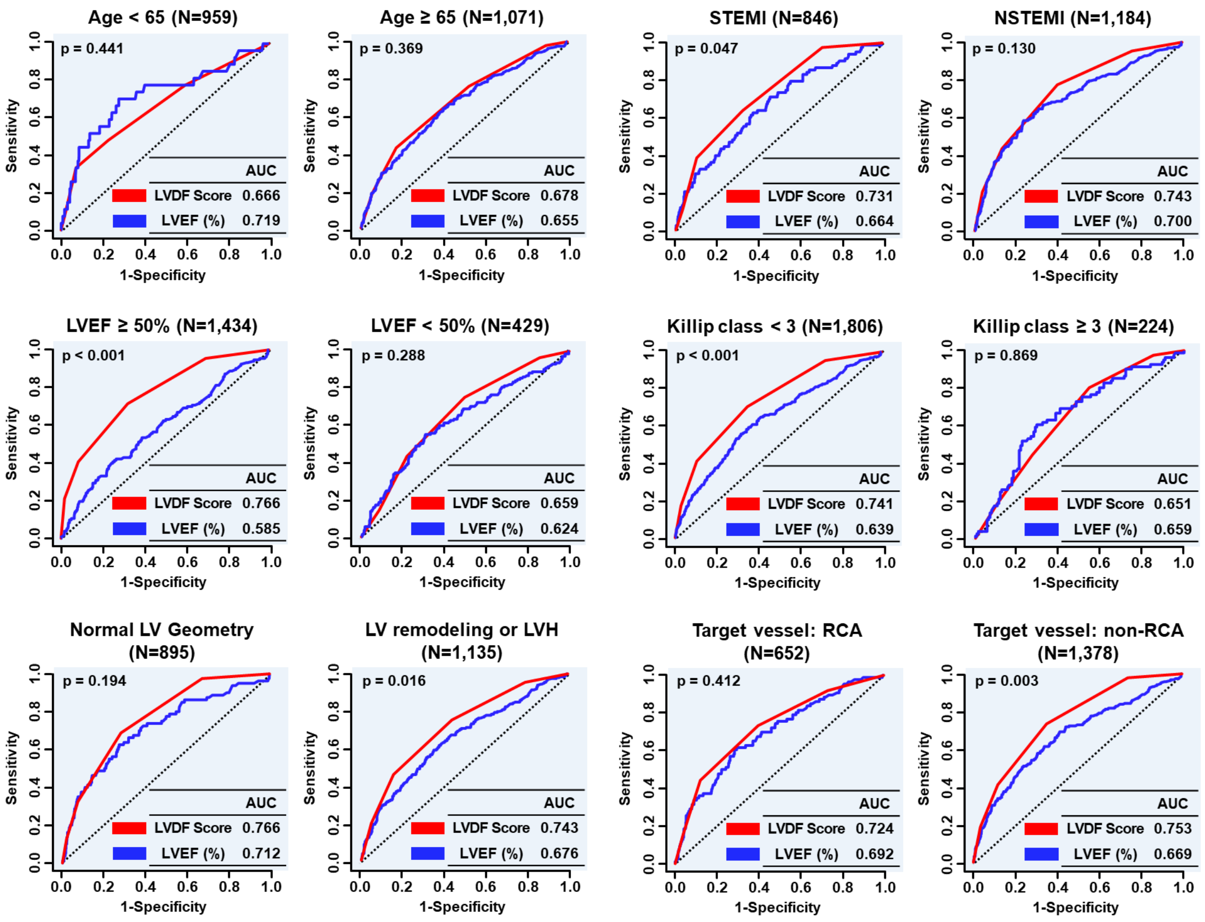

LVEF, left ventricular ejection fraction; LVH, left ventricular hypertrophy; NSTEMI, non ST-segment elevation myocardial infarction; STEMI, ST-segment elevation myocardial infarction; RCA, right coronary artery.

Supplementary Figure 4. Comparison of the predictive performance of the individual LVDF parameters and LVEF for all-cause death and hospitalization due to HF

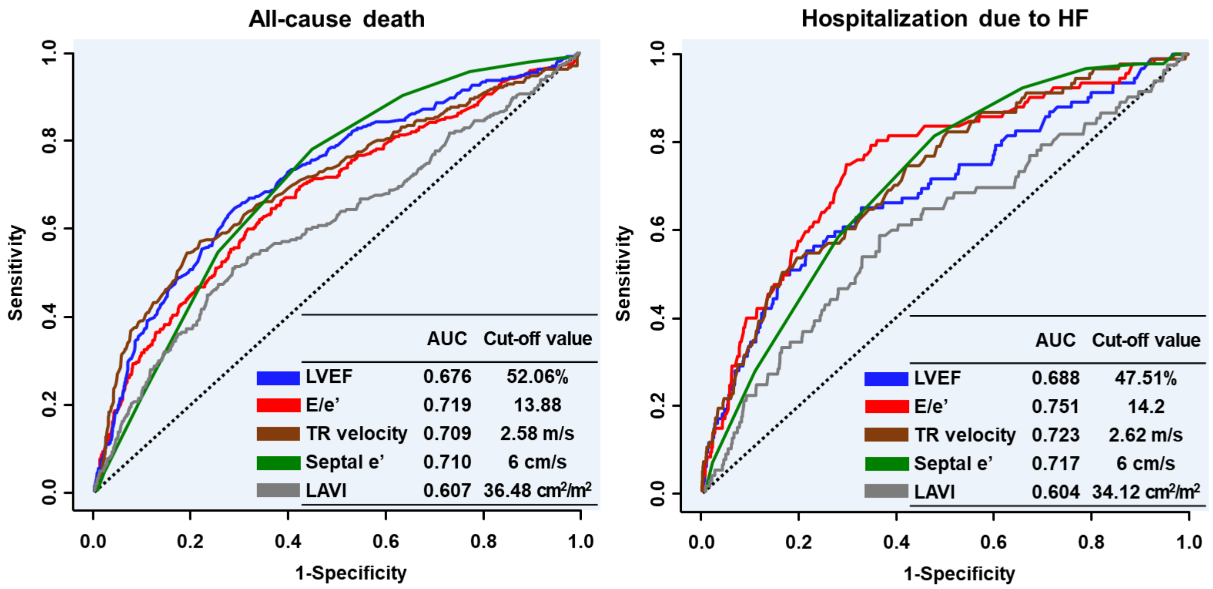

HF, heart failure; LAVI, left atrial volume index; LVEF, left ventricular ejection fraction; TR, tricuspid regurgitation.

Supplementary Figure 5. Subgroup analysis for factors that could affect septal e'

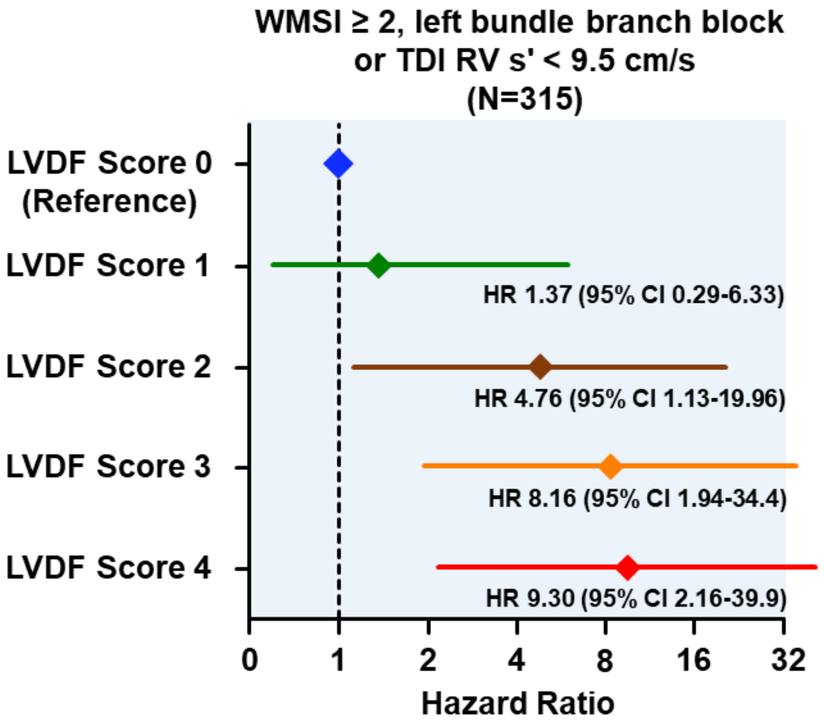

CI, confidence interval; HR, hazard ratio; LVDF, left ventricular diastolic function; RV, right ventricle; TDI, tissue doppler imaging; WMSI, wall motion score index.
